# Differential effects of high dose omega-3 fatty acids on metabolism and inflammation in patients with obesity: eicosapentaenoic and docosahexaenoic acid supplementation

**DOI:** 10.3389/fnut.2023.1156995

**Published:** 2023-05-05

**Authors:** Angélica Borja-Magno, Martha Guevara-Cruz, Adriana Flores-López, Silvia Carrillo-Domínguez, Julio Granados, Clorinda Arias, Mary Perry, Barry Sears, Hector Bourges, F. Enrique Gómez

**Affiliations:** ^1^Departamento de Fisiología de la Nutrición, Instituto Nacional de Ciencias Médicas y Nutrición Salvador Zubirán, Mexico City, Mexico; ^2^Servicio de Nutriología Clínica, Instituto Nacional de Ciencias Médicas y Nutrición Salvador Zubirán, Mexico City, Mexico; ^3^Departamento de Nutrición Animal, Instituto Nacional de Ciencias Médicas y Nutrición Salvador Zubirán, Mexico City, Mexico; ^4^Departamento de Trasplantes, Instituto Nacional de Ciencias Médicas y Nutrición Salvador Zubirán, Mexico City, Mexico; ^5^Departamento de Medicina Genómica y Toxicología Ambiental, Instituto de Investigaciones Biomédicas, Universidad Nacional Autónoma de México, Mexico City, Mexico; ^6^Inflammation Research Foundation, Peabody, MA, United States; ^7^Dirección de Nutrición, Instituto Nacional de Ciencias Médicas y Nutrición Salvador Zubirán, Mexico City, Mexico

**Keywords:** obesity, inflammation, metabolism, omega-3 fatty acids, eicosapentaenoic (EPA), docosahexaenoic (DHA), nutrition

## Abstract

**Background:**

Obesity is complicated by low-grade chronic inflammation characterised by increases in inflammatory proteins and cells in peripheral blood. It has been known that omega-3 fatty acids (FA) like eicosapentaenoic (EPA) and docosahexaenoic (DHA) could modulate the inflammatory process and improve metabolic markers.

**Objective:**

This study aimed to determine the effect of high-dose omega-3 FA on metabolic and inflammatory markers among patients with obesity and healthy volunteers.

**Methods:**

This prospective study included 12 women with obesity (body mass index [BMI] ≥ 35.0 kg/m^2^) and 12 healthy women (BMI < 24.0 kg/m^2^) who were supplemented with a dose of 4.8 g/day (3.2 g EPA plus 1.6 g DHA) for 3 months followed by no treatment for 1 month. Plasma metabolic and inflammatory markers and levels of mRNA transcripts of CD4^+^ T lymphocyte subsets were determined monthly.

**Results:**

None of the participants exhibited changes in weight or body composition after study completion. EPA and DHA supplementation improved metabolic (insulin, Homeostatic Model Assessment of Insulin Resistance [HOMA-IR], triglyceride [TG]/ high-density lipoprotein [HDL] ratio, TG, and arachidonic acid [AA]/EPA ratio) and tumor necrosis factor-alpha (TNF-α). Moreover, the levels of mRNA transcripts of T CD4^+^ lymphocyte subsets (TBX21, IFNG, GATA-3, interleukin [IL]-4, FOXP3, IL-10 IL-6, and TNF-α), were down-regulated during the intervention phase. After 1 month without supplementation, only insulin, HOMA-IR and the mRNA transcripts remained low, whereas all other markers returned to their levels before supplementation.

**Conclusion:**

Supplementation with high-dose omega-3 FAs could modulate metabolism and inflammation in patients with obesity without weight loss or changes in body composition. However, these modulatory effects were ephemeral and with clear differential effects: short-duration on metabolism and long-lasting on inflammation.

## Introduction

Obesity has been described to be associated with a chronic state of low-grade inflammation characterised by high levels of inflammatory proteins like leptin, interleukin(IL)-6, IL-8 and tumor necrosis factor-alpha (TNF-α) among others (1). In addition, mild increases have been reported in total white blood cells (leukocytosis) mostly of the T cell lineage, partially due to the T CD4+ helper (Th) lymphocyte subsets. These cells are important regulators of the inflammatory process: Th2 and regulatory T cells (Treg) have anti-inflammatory activities, whereas the Th1 and Th17 cells have pro-inflammatory function (2, 3). Altogether, these inflammatory proteins and cells play an important role in the development of insulin resistance, diabetes mellitus and metabolic syndromes (4).

Nutrients including omega-3 fatty acids (FAs) such as eicosapentaenoic (EPA) and docosahexaenoic (DHA) acids have shown anti-inflammatory effects (5, 6). Evidence-based findings have shown that omega-3 FAs could attenuate CD4+ T lymphocyte activation (7) and migration to adipose tissue (8). *In vitro* studies showed that T cells treated with EPA and DHA manifested lower cytokine synthesis and reduced Th1 differentiation (9). However, the effects of omega-3 FA supplementations on inflammatory markers remain controversial. The anti-inflammatory effect has been shown in human plasma levels of TNF-α, IL-6, and C-reactive protein (CRP) (5, 10). However, other reports showed no anti-inflammatory effects (11) or reduction in cardiovascular events (12). To date, studies on the effects of EPA and DHA *in vivo* on CD4+ T cell subsets in patients with obesity are scarce (8, 9).

Therefore, we evaluated the effects of supplementation with high-dose omega-3 FA, EPA plus DHA, for 3 months and 1 month without supplementation on metabolic and inflammatory markers in patients with obesity and healthy volunteers.

## Participants and methods

We evaluated 24 Mexican women between 25 and 45 years of age: 12 women with a body mass index [BMI] ≥ 35.0 (obesity) and 12 healthy volunteers with BMI < 24.0 (control). The exclusion criteria were presence of diabetes mellitus, hypertension, liver or kidney diseases, or pregnancy or receiving any medication or dietary supplements. This study was approved by the Ethics Committee of the INCMNSZ and all participants signed an informed consent. This study is registered at Clinical trials with the ID NCT05219890.

### Study design

**Figure 1:**
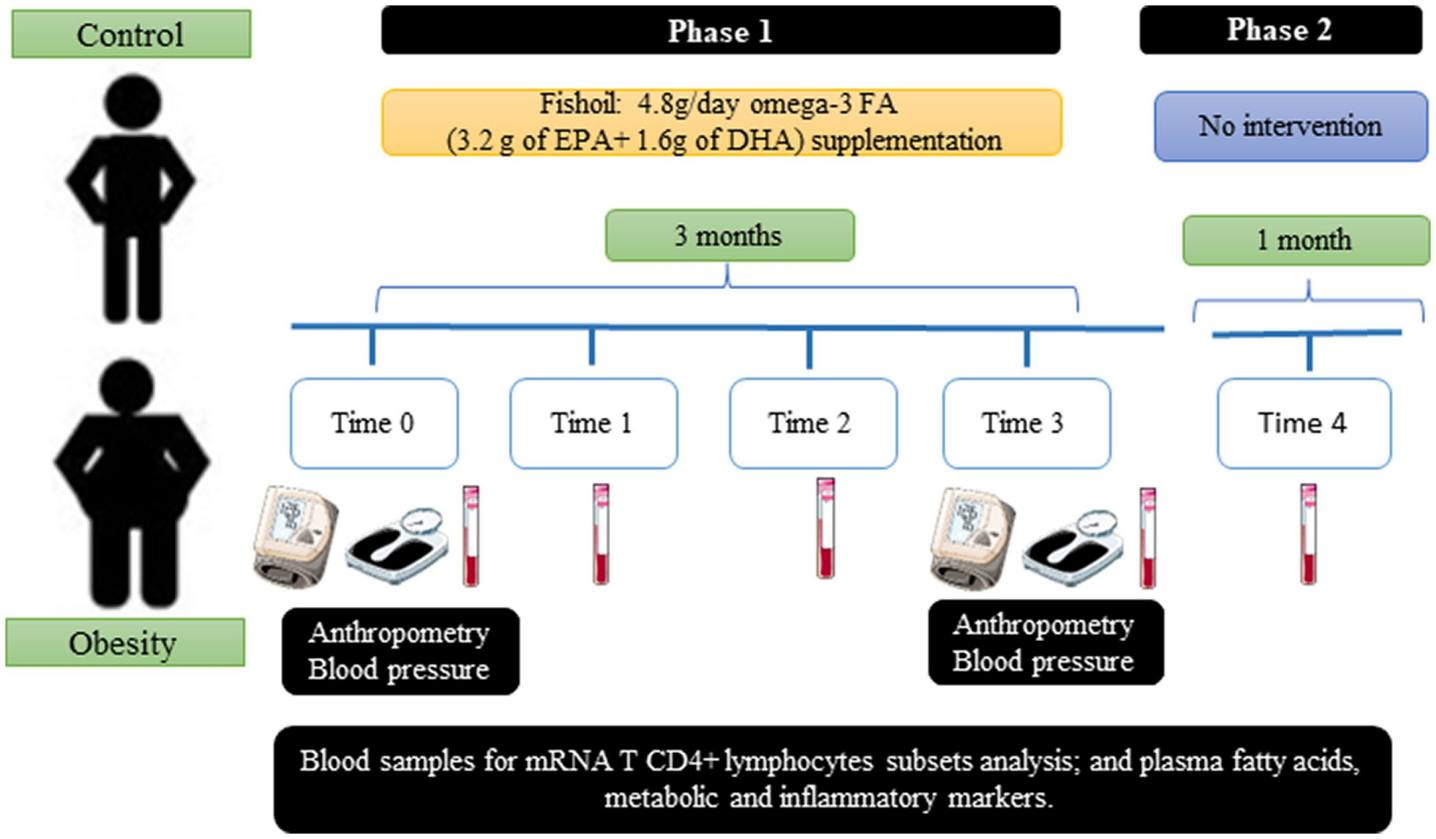
Study design.

This was a prospective, experimental, open-label (non-blinded), and comparative study to evaluate the effects of omega-3 FAs (EPA and DHA) supplementation on metabolic and inflammatory markers. The study comprised two phases: the first phase consisted of a 3 month supplementation with fish oil (times 0, 1, 2, and 3) and the second one included one month without supplementation (time 4) (Figure 1). At times 0 and 3, blood pressure and anthropometric measurements were registered. None of the participants were asked to change their diet or daily physical activity (increase or decrease). Furthermore, none of the participants received nutritional advice of any type.

### Fish oil supplement

Omega-3 FA supplementation was performed using fish oil capsules provided by the Inflammation Research Foundation (Peabody, MA, United States). Each 1 gram capsule contained 0.6 g of omega-3 FA (0.4 g of EPA and 0.2 g of DHA). Therefore, to provide 4.8 g of omega-3 FA, each participant received 8 capsules per day, distributed as follows: 2 capsules at breakfast, 4 capsules supplemented with the main meal, and 2 capsules at dinner. This dose selection was based on previous research revealing increases in plasma concentration of EPA and DHA at maximum level, similar to doses up to 7 g/day (13).

Each participant received 2 bottles (120 capsules per bottle, a total of 240 capsules enough to cover the dosage for 1 month) of fish oil supplement at the time of recruitment (time 0). After 1 month (time 1), 2 new bottles of supplement were provided for the following month (time 2). This was repeated for the last month (time 3). To determine the duration of the lasting effects of the fish oil, we included a 1 month period without supplementation (time 4).

Compliance was determined by requesting all participants to bring the bottles of supplement at the end each month, with the remaining capsules (if any). The study staff always kept in touch with the participants by phone messages for monitoring tolerance to the supplement.

### Anthropometry, body composition, and blood pressure

Height and weight were used to calculate BMI using the formula [weight (kg)/height (m)^2^] (14). Body composition was determined by electric bioimpedance using a Full Body Composition X-Contact 356 Analyzer (Jawon Medical, Seoul, South Korea), to quantify the fat and fat-free (lean) masses, which was performed at times 0 and 3.

### Plasma metabolic and inflammatory markers

Peripheral blood samples were collected in the morning after 10 h of fasting, in vacutainer tubes with EDTA-K_2_ anticoagulant. Total leukocytes were counted using Neubauer chambers. Erythrocyte sedimentation rate (ESR) was measured by a standard method. Blood plasma obtained after centrifugation of a small sample was stored at −80°C until assayed.

Plasma glucose, total cholesterol, high density cholesterol (HDL-C), low density cholesterol (LDL-C), and triglycerides (TG) levels were quantified using a Cobas c111 analyzer (Roche, Indianapolis, IN, United States). The TG/HDL-C ratio (reference value <3.0) was calculated (15). Insulin levels were determined using an ELISA kit (ALPCO, Salem, NH, United States). The HOMA-IR index (reference value < 2.5) was used as an indicator of insulin resistance (16).

Plasma fatty acids were extracted with chloroform/methanol and derivatised to their methyl esters (FAME) with methanol in boron trichloride. The FAMEs were quantified with a Varian 3,400 gas chromatograph using tridecanoic acid (13:0) as internal standard (17).

The following proteins were quantified by multiplexed immunoassay using the MAGPIX luminometer (Luminex, Austin, Texas, United States): leptin (L; reference value ≤ 15.7 ng/mL) (18) TNF-α, IL-6, IL-8, IL-10 and transforming grow factor-beta (TGF-β). Total adiponectin (A) was quantified using an ELISA kit (ALPCO, Salem, NH, United States) and the A/L ratio calculated (reference value > 1.0) (19). Plasma resolvin E1 (RvE1) was quantified using a competitive ELISA kit (MyBiosource, San Diego, CA, United States).

### mRNA expression in the CD4^+^ T lymphocyte subsets (Th1, Th2, and Treg cells)

The level of transcription of genes expressed in T lymphocyte subsets (Th1, Th2, and Treg) was determined by reverse-transcription quantitative polymerase chain reaction (RT-PCR). First, peripheral blood mononuclear cells (PBMC) were isolated by Lymphoprep™ density gradient centrifugation (Stemcell technologies, Vancouver, Canada). Total RNA was extracted from the PBMC (without further separation of monocytes or T cell subsets) with the TRIZOL reagent (Invitrogen Life Technologies, Carlsbad CA, United States) and isopropanol precipitation (20). RNA integrity was determined in 1% agarose gel recipe by electrophoresis. Additionally, RNA concentration was measured by ultraviolet absorbance spectrophotometry at 260 nm. Complementary DNA (cDNA) was synthesised from 1 μg of total RNA with Moloney Murine Leukemia Virus reverse transcriptase (Invitrogen, Carlsbad, California, United States), oligo-dT_15_ primer (Invitrogen, Groningen, Holland), RNase inhibitor (Applied Biosystems, Warrington, United Kingdom), a mixture of dNTP’s (Invitrogen, Carlsbad, California, United States), 5X RT-buffer (Invitrogen, Carlsbad, CA, United States), and nuclease-free water (Sigma-Aldrich, St. Louis, MO, United States) (20, 21). The relative expression of the mRNA was determined by RT-PCR in 2 μL of cDNA with 10 μL of 2X SYBR green Master mix (Roche Diagnostics, Lewis, United Kingdom) and 2 μL of the forward and reverse mixture of primers at 10 mM final concentration in a RotorGene Q thermocycler (QIAGEN, Germany). The level of transcription of IL-6, TNF-α was measured. In addition, the level of transcription of the following genes of the CD4^+^ T lymphocyte subsets was determined: T-box transcription factor 21 (TBX21) and IFNG in Th1 cells, GATA3 and IL-4 in Th2 cells, and forkhead box P3 (FOXP3) and IL-10 in Treg cells. Furthermore, 18S-ribosomal rRNA was used as the housekeeping gene (22) (Supplementary Table S1). The level of transcription of the mRNAs was determined with the ΔΔ*Ct* method (23).

### Statistical analysis

Continuous variables were expressed as mean ± standard deviation (SD). The difference between groups at time 0 were analyzed using Student’s *t*-test for independent groups. One-way analysis of variance (ANOVA) was used for the analysis of gene expression pattern over time. Analysis of plasma metabolic and inflammatory markers was performed by two-way ANOVA for repeated measures to compare the effect of omega-3 FA supplementation between the groups and time. The Bonferroni post-hoc test was used to identify significant differences between times. Data was analyzed using Statistical Package for Social Science, version 20 (SPSS Inc., Chicago, IL, United States) and GraphPad Prism 8. Statistical significance was set to *p* < 0.05.

## Results

### Population study

**Figure 2:**
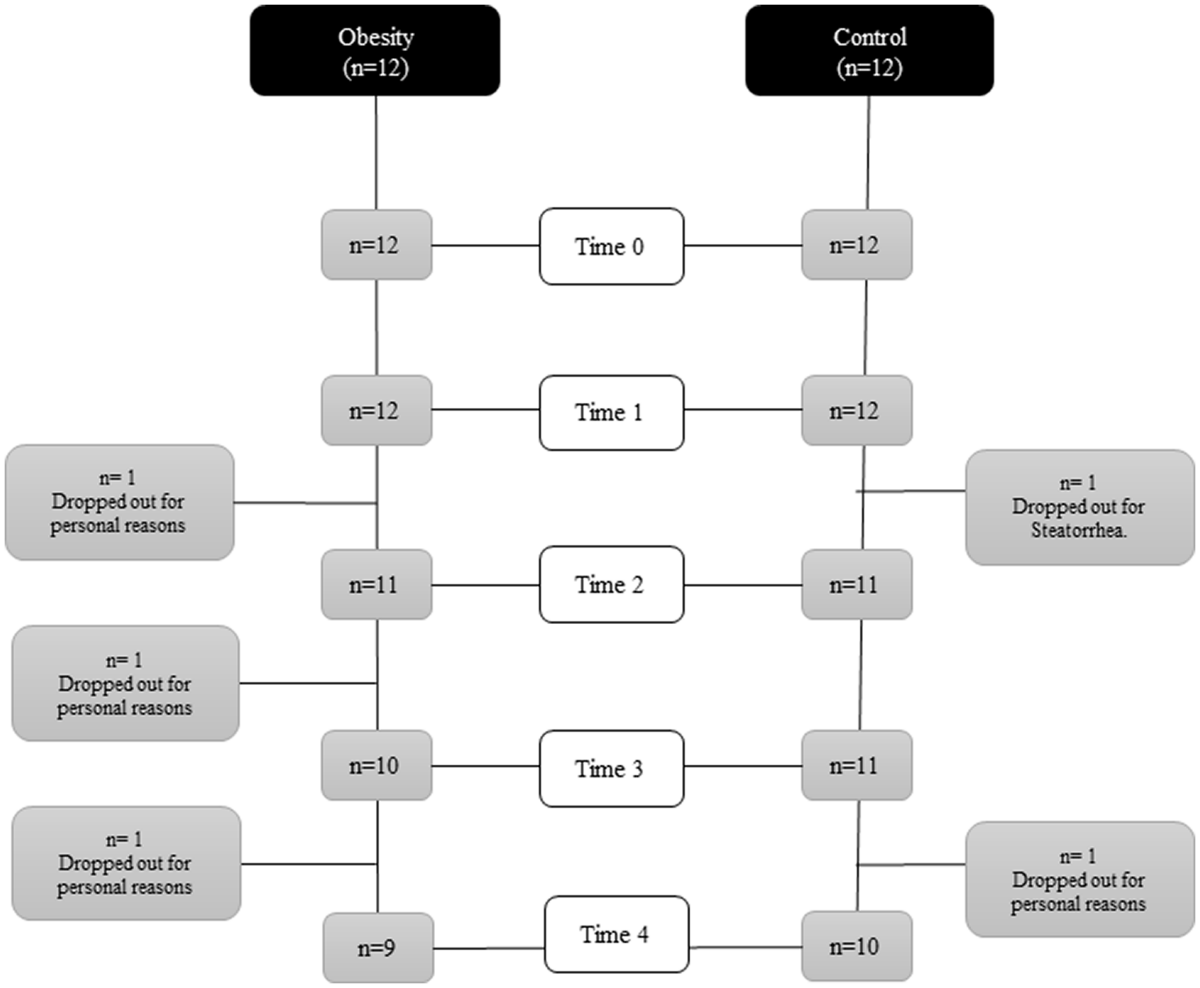
Flowchart of the course of the study.

A flowchart is shown in Figure 2. All anthropometric measurements, except for height, were significantly higher (*p* < 0.001) in the group with obesity: weight, BMI, fat mass, and lean body mass. Regarding metabolic markers, the group with obesity showed significantly elevated blood pressure (BP; systolic (*p* < 0.001) and diastolic (*p* < 0.02)); higher glucose (*p* < 0.02), insulin (*p* < 0.001), HOMA-IR (*p* < 0.001), TG (*p* < 0.001), and TG/HDL-C ratio (*p* < 0.02); and lower levels of HDL-C (*p* < 0.02) (Table 1) compared with the control. Regarding inflammatory markers, the group with obesity had significantly higher total leukocytes (*p* < 0.005), ESR (*p* < 0.001), and leptin (*p* < 0.01) and lower total adiponectin and A/L ratio (*p* < 0.05) (Table 1) compared with the healthy participants. The following metabolic and inflammatory markers were different between the groups, without reaching statistical significance: LDL-C, IL-6, IL-8, and IL-10 were higher, whereas TGF-β was lower (Table 1). Total cholesterol and TNF-α were identical between both groups at baseline.

**Table 1:** Anthropometric, metabolic and inflammatory characteristics of the participants at the beginning of the study (Time 0).

	Control	Obesity	*p* value
	(*n* = 12)	(*n* = 12)	
Anthropometry and body composition			
Age (years) mean ± SD	31.7 ± 6.7	38.1 ± 6.0	<0.05
Median	29.5	39	
Range	24–43	27–45	
Height (m) mean ± SD	1.62 ± 0.05	1.60 ± 0.08	0.18
Median	1.63	1.56	
Range	1.55–1.70	1.51–1.75	
Weight (kg) mean ± SD	56.3 ± 9.0	102.1 ± 15.4	<0.001
Median	55.7	103.3	
Range	49.1–63.6	82.7–131.0	
BMI (kg/m^2^) mean ± SD	21.7 ± 2.2	39.8 ± 5.5	<0.001
Median	21.4	36.7	
Range	17.8–23.9	35.0–52.7	
Fat mass (kg) mean ± SD	15.0 ± 3.9	42.7 ± 7.8	<0.001
Median	15.1	39.6	
Range	8.3–20.8	34.5–60.4	
Fat mass (%) mean ± SD	25.5 ± 5.1	41.9 ± 3.6	<0.001
Median	24.3	42.6	
Range	15.0–34.9	36.5–47.6	
Lean body mass (kg) mean ± SD	41.8 ± 3.6	59.3 ± 9.8	<0.001
Median	41.4	57.9	
Range	37.5–49	46.8–76.6	
Lean body mass (%) mean ± SD	74.4 ± 5.3	58.1 ± 3.6	<0.001
Median	76.2	57.1	
Range	65.1–85.0	52.4–63.5	
Metabolic markers^§^			
Systolic BP (mm Hg) mean ± SD	106 ± 8	123 ± 8	<0.001
Median	107	126	
Range	95–125	109–136	
Diastolic BP (mm Hg) mean ± SD	67 ± 6	78 ± 9	<0.02
Median	65	79	
Range	58–82	63–92	
Glucose (mg/dL) mean ± SD	82.4 ± 7.5	93.3 ± 11.8	<0.02
Median	81.7	89.5	
Range	73.1–99.2	80.8–112.6	
Insulin (mU/L) mean ± SD	3.2 ± 1.1	13.0 ± 8.8	<0.001
Median	3.6	10.7	
Range	1.7–4.6	4.5–28.7	
HOMA-IR mean ± SD	0.7 ± 0.2	3.2 ± 2.3	<0.001
Median	0.7	3.7	
Range	0.3–1.0	0.9–8.0	
Total cholesterol (mg/dL) mean ± SD	164.0 ± 31.1	167.0 ± 39.1	0.88
Median	167	159	
Range	120.1–215.0	114.2–236.6	
LDL-C (mg/dL) mean ± SD	85.9 ± 31.4	108.9 ± 34.4	0.19
Median	81.5	112.7	
Range	32.0–137.8	60.6–157.3	
HDL-C (mg/dL) mean ± SD	42.2 ± 8.9	33.0 ± 9.9	<0.02
Median	41.9	30.8	
Range	29.1–57.9	18.7–52.2	
Triglycerides (mg/dL) mean ± SD	89.4 ± 37.8	191.0 ± 102.1	<0.001
Median	77.1	159.8	
Range	38.1–130.0	79.2–454.1	
TG/HDL-C ratio mean ± SD	2.2 ± 1.1	6.7 ± 5.9	<0.02
Median	1.96	5.5	
Range	0.8–4.6	2.5–24.3	
Inflammatory markers^‡^			
Leukocytes (x10^3^/mm^3^) mean ± SD	5.7 ± 0.87	8.1 ± 1.9	<0.005
Median	5.4	8.6	
Range	4.4–7.2	4.5–10.1	
ESR (mm/h) mean ± SD	6 ± 4	18 ± 12	<0.001
Median	5	19	
Range	2–15	Jan-37	
IL-6 (pg/mL) mean ± SD	0.9 ± 0.9	2.2 ± 3.6	0.63
Median	0.93	0.9	
Range	0.01–1.7	0.1–12.3	
IL-8 (pg/mL) mean ± SD	0.9 ± 0.6	2.7 ± 3.0	0.71
Median	0.64	1.1	
Range	0.64–2.50	0.4–9.8	
TNF-α (pg/mL) mean ± SD	1.7 ± 0.5	1.9 ± 0.9	0.75
Median	1.7	1.9	
Range	0.6–2.6	0.7–3.6	
IL-10 (pg/mL) mean ± SD	2.9 ± 4.8	4.0 ± 5.5	0.66
Median	1.2	1.6	
Range	0.06–13.8	0.3–17.5	
TGF-β (ng/mL) mean ± SD	5.2 ± 4.5	4.0 ± 4.2	0.63
Median	3.4	2.5	
Range	1.8–17.2	0.8–15.3	
Leptin (ng/mL) mean ± SD	9.6 ± 7.0	35.5 ± 25.4	<0.01
Median	8.3	23.3	
Range	1.0–26.9	8.2–77.7	
Adiponectin (μg/mL) mean ± SD	7.5 ± 2.5	4.5 ± 1.7	<0.05
Median	7.3	4.1	
Range	4.7–11.0	2.2–7.3	
A/L ratio mean ± SD	1.6 ± 2.1	0.3 ± 0.3	<0.05
Median	0.8	0.2	
Range	0.3–7.4	0.1–0.8	

^§^Reference values: systolic BP < 120 mm Hg, diastolic BP < 80 mm Hg, glucose < 100 mg/dL, total cholesterol < 200 mg/dL, HDL-C > 40 mg/dL, LDL-C < 100 mg/dL, TG < 150 mg/dL, TG/HDL ratio < 3.0 (14). HOMA-IR < 2.5 (15), Insulin < 25 mU/L. ^‡^Reference values: leukocytes < 10.0×10^3^/mm^3^, ESR < 20 mm/h, leptin < 15.7 ng/mL (17) A/L ratio > 1.0 (18). Mean ± SD. Student’s *t*-test for independent groups. Statistical significance *p* < 0.05. BP, blood pressure. TG/HDL-C ratio, triglycerides to high density lipoprotein cholesterol ratio. ESR, erythrocyte sedimentation rate. A/L ratio, adiponectin to leptin ratio.

**Figure 3:**
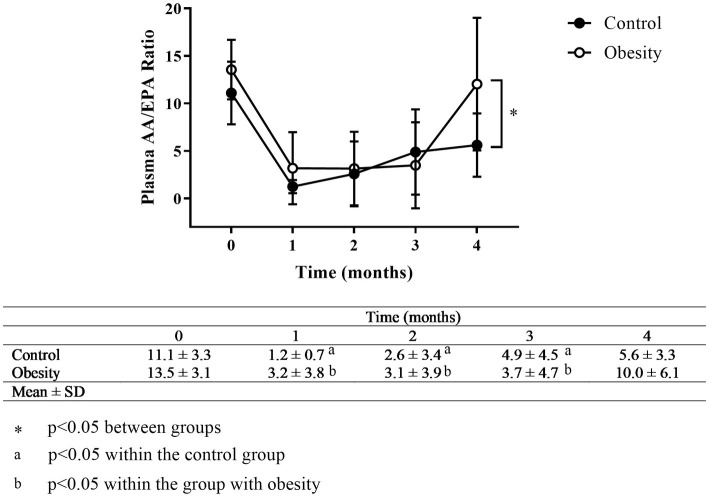
Time-course of the AA/EPA ratio. Data shown in means ± SD.

The AA/EPA ratio in the control and obesity groups was 11.1 and 13.6, respectively (Figure 3). In addition, omega-6/omega-3 ratio in the control and obesity groups was 12.4 and 10.3 (Supplementary Table S3), respectively, which indicated low consumption of omega-3 FA.

**Figure 4:**
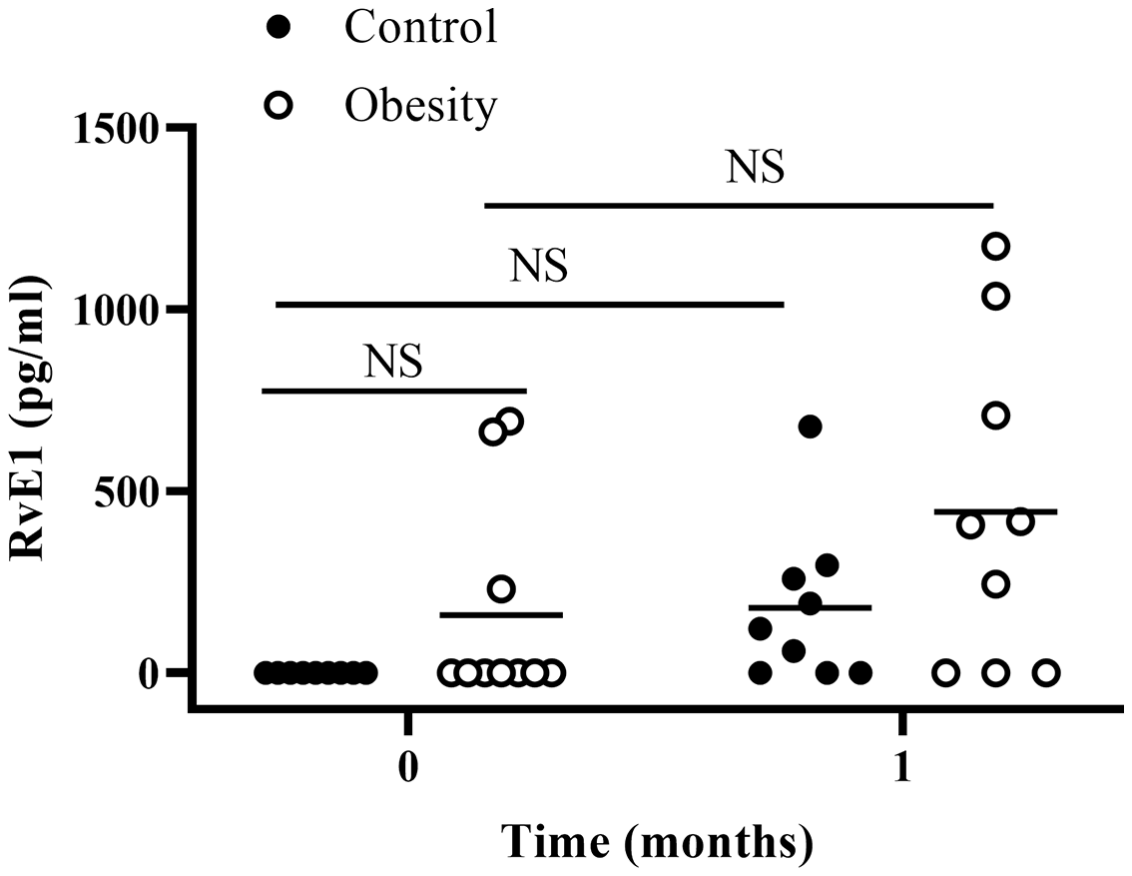
Plasma RvE1 at the beginning of the study (time 0) and after 1 month of fish oil supplementation. Horizontal bars represent mean value. NS: non-statistically significant.

Plasma RvE1, a specialised pro-resolving mediator derived from EPA, was undetectable in the control group (the assay sensitivity was 1 pg/mL). However, it was detected only in three patients in the group with obesity (average of 159 pg/mL) at baseline (Figure 4).

### Effect of omega-3 FA supplementation on metabolic and inflammatory markers

Regardless of their nutritional status, none of the participants exhibited changes in body weight or body composition after the 3 months of supplementation (Supplementary Table S2).

Therefore, the effects of fish oil supplementation, reported here, were only due to the effect of omega-3 FA. For clarity, the main effects of omega-3 FA supplementation on metabolic and inflammatory markers are described in each group.

### Control group

At the beginning of the study all metabolic and inflammatory markers were within normal and healthy values (Table 1). Omega-3 FA supplementation resulted in changes in some of the markers as early as 1 month of supplementation, and always towards a better health status. Of note, the metabolism of glucose and lipids improved as fasting insulin and HOMA-IR significantly decreased, whereas total cholesterol and TG decreased, and HDL-C increased without statistical significance (Table 2). Similarly, TNF-α decreased with supplementation. However IL-6 and leptin non-significantly decreased (Table 2). Markers of wellness including AA/EPA (Figure 3) and omega-6/omega-3 ratios (Supplementary Table S3) improved during the intervention. Other markers of wellness such as A/L and TG/HDL-C (Table 2) decreased without statistical significance. Moreover, plasma RvE1, which was undetectable at time 0, non-significantly increased to an average of 179 pg/mL after supplementation (median: 122 pg/mL) (Figure 4).

**Table 2:** Time-course effect of omega-3 fatty acid supplementation (EPA and DHA) on metabolic and inflammatory markers.

		Time 0	Time 1	Time 2	Time 3	Time 4	*p* value^†^	*p* value^₸^	*p* value^¥^
Metabolic markers									
Glucose (mg/dL)	Control	82.4 ± 7.5	82.0 ± 8.0	74.4 ± 15.7	83.3 ± 7.8	82.5 ± 8.1	0.33	0.08	0.02
	Obesity	93.3 ± 11.8	88.9 ± 10.8	92.4 ± 7.7	91.9 ± 8.7	86.2 ± 8.0			
Insulin (mU/L)	Control	3.2 ± 1.1	1.7 ± 1.1	2.4 ± 1.3	2.2 ± 2.1	2.5 ± 1.5	0.01	0.001	0.07
	Obesity	13.0 ± 8.8	6.9 ± 3.4*	7.1 ± 3.9*	8.4 ± 4.5	7.4 ± 2.8*			
HOMA-IR	Control	0.7 ± 0.2	0.4 ± 0.2	0.5 ± 0.3	0.5 ± 0.5	0.5 ± 0.3	0.01	0.001	0.01
	Obesity	3.2 ± 2.3	1.5 ± 0.9*	1.5 ± 1.0*	1.8 ± 1.1	1.5 ± 0.7*			
Total cholesterol (mg/dL)	Control	164.0 ± 31.1	150.3 ± 30.3	137.3 ± 32.1	152.4 ± 25.5	148.2 ± 31.4	0.11	0.26	0.56
	Obesity	167.0 ± 39.1	165.7 ± 28.3	160.9 ± 33.0	166.9 ± 31.2	173.9 ± 46.7			
LDL-C (mg/dL)	Control	85.9 ± 31.4	94.1 ± 35.8	82.2 ± 29.8	88.0 ± 33.9	95.2 ± 37.9	0.24	0.11	0.62
	Obesity	108.9 ± 34.4	115.7 ± 27.8	112.6 ± 31.8	117.9 ± 32.3	112.3 ± 40.9			
HDL-C (mg/dL)	Control	42.2 ± 8.9	50.5 ± 12.7	49.8 ± 18.1	57.2 ± 19.8	47.9 ± 13.2	0.08	0.01	0.17
	Obesity	33.0 ± 9.9	36.8 ± 9.1	33.9 ± 10.3	36.6 ± 14.0	36.4 ± 12.3			
Triglycerides (mg/dL)	Control	89.4 ± 37.8	74.4 ± 29.1	78.2 ± 46.2	83.2 ± 45.1	86.5 ± 42.9	0.24	0.01	0.09
	Obesity	191.0 ± 102.1	148.4 ± 58.7	149.2 ± 76.6	146.1 ± 60.4	208.2 ± 125.1			
TG/HDL-C ratio	Control	2.2 ± 1.1	1.6 ± 0.7	1.5 ± 0.9	1.7 ± 1.1	2.0 ± 1.2	0.37	0.001	0.20
	Obesity	6.7 ± 5.9	4.3 ± 2.1	4.7 ± 3.6	4.7 ± 3.3	6.6 ± 5.8			
Inflammatory markers									
Leukocytes (x10^3^/mm^3^)	Control	5.7 ± 0.8	5.7 ± 1.4	6.3 ± 1.9	5.8 ± 1.7	5.3 ± 1.3	0.84	0.001	0.97
	Obesity	8.1 ± 1.9	8.1 ± 1.5	8.1 ± 1.7	7.9 ± 1.8	7.5 ± 1.4			
ESR (mm/h)	Control	6 ± 4	9 ± 7	6 ± 4	9. ± 9	10 ± 6	0.45	0.001	0.93
	Obesity	16 ± 12	17 ± 11	17 ± 11	19 ± 14	22 ± 13			
IL-6 (pg/mL)	Control	0.9 ± 0.9	0.2 ± 0.4	0.1 ± 0.1	0.4 ± 0.3	1.0 ± 1.1	0.47	0.07	0.72
	Obesity	2.2 ± 3.6	1.0 ± 1.3	1.5 ± 2.1	1.6 ± 2.9	1.9 ± 1.9			
IL-8 (pg/mL)	Control	0.9 ± 0.6	0.6 ± 0.7	0.6 ± 0.4	1.0 ± 0.7	1.0 ± 0.6	0.38	0.01	0.27
	Obesity	2.7 ± 3.0	2.4 ± 1.9	2.4 ± 2.7	3.8 ± 2.7	2.6 ± 1.3			
TNF-α (pg/mL)	Control	1.7 ± 0.5	0.8 ± 0.6 *	0.9 ± 0.6 *	1.0 ± 0.4*	1.8 ± 0.9	0.01	0.24	0.32
	Obesity	1.9 ± 0.9	1.1 ± 0.5 *	1.3 ± 0.7 *	1.7 ± 1.0	1.8 ± 0.8			
IL-10 (pg/mL)	Control	2.9 ± 4.8	2.1 ± 1.8	2.7 ± 2.4	3.1 ± 3.2	2.7 ± 2.9	0.90	0.25	0.45
	Obesity	4.0 ± 5.5	9.0 ± 14.8	5.2 ± 8.0	7.4 ± 10.6	9.4 ± 14.7			
TGF-β (ng/mL)	Control	5.2 ± 4.5	4.2 ± 2.8	5.6 ± 6.4	5.6 ± 5.0	4.2 ± 3.6	0.06	0.43	0.99
	Obesity	4.0 ± 4.2	5.0 ± 2.5	6.6 ± 4.5	7.4 ± 8.8	2.7 ± 2.4			
Leptin (ng/mL)	Control	9.6 ± 7.0	4.1 ± 3.5	5.8 ± 5.1	5.7 ± 6.2	5.8 ± 4.8	0.53	0.03	0.02
	Obesity	35.5 ± 25.4	19.2 ± 5.3	19.9 ± 9.5	25.2 ± 16.1	20.7 ± 20.0			
Adiponectin (μg/mL)	Control	7.5 ± 2.5	8.5 ± 2.2	7.2 ± 2.4	8.5 ± 3.1	—	0.07	0.057	0.08
	Obesity	4.5 ± 1.7	4.7 ± 2.6	4.2 ± 1.9	4.6 ± 2.6	—			
A/L ratio	Control	1.6 ± 2.1	3.9 ± 3.9	6.6 ± 13.7	3.3 ± 2.8	—	0.62	0.002	0.49
	Obesity	0.3 ± 0.3	0.2 ± 0.1	0.2 ± 0.1	0.3 ± 0.2	—			

Mean ± SD. Two-way ANOVA. ^†^ Time factor (comparisons within groups vs. time 0). ^₸^ Group factor (comparison between groups obesity vs control). ^¥^ Interaction (effect of group and time on the metabolic or inflammatory marker). * Statistical significance *p* < 0.05. — not determined. TG/HDL-C ratio, triglycerides to high density lipoprotein cholesterol ratio; ESR, erythrocyte sedimentation rate; A/L, adiponectin to leptin ratio.

#### Group with obesity

After one month of supplementation, insulin (*p* < 0.01), HOMA-IR (*p* < 0.01), and TNF-α (*p* < 0.01), were significantly decreased, and remained low within the duration of the study, except for TNF-α which returned to its initial value at time 3 (Table 2). On the other hand, there were non-significant reductions in TG, leptin, or IL-6 levels, with non-significant increases in IL-10 and TGF-β. None of the following markers changed during supplementation: systolic and diastolic BP, glucose, cholesterol, HDL-C, LDL-C, total leukocytes, ESR, and IL-8 (Table 2). Markers of wellness such as AA/EPA (Figure 3) and omega-6/omega-3 ratios (Supplementary Table S3) decreased. However, the TG/HDL-C ratio decreased without statistical significance during the supplementation period, without change in the A/L ratio (Table 2) with supplementation. No changes were observed in RvE1 levels after supplementation, from 159 pg/mL at time 0 to 443 pg/mL (Figure 4).

#### Effect of omega-3 FA supplementation on mRNA of CD4+ T lymphocyte subsets (Th1, Th2, and Treg cells)

**Figure 5:**
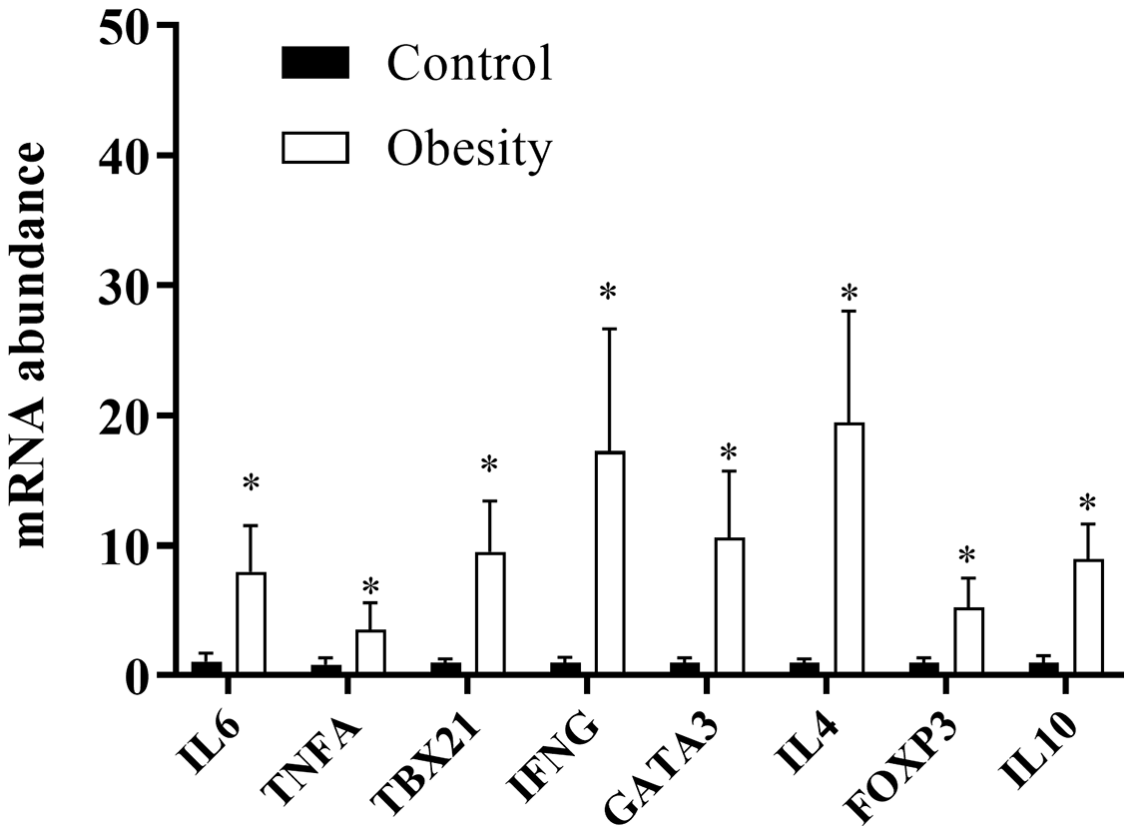
mRNA levels in T CD4+ lymphocyte subsets at the beginning of the study. Data shown in means ± SEM.

**Figure 6:**
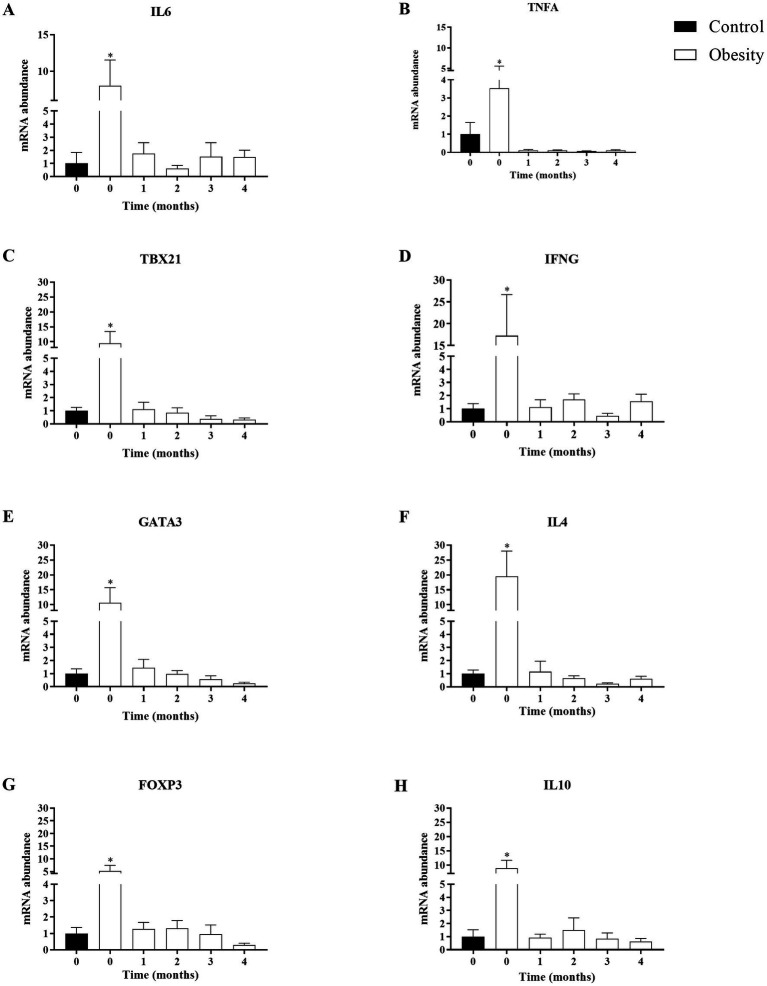
Time-course effect of omega-3 fatty acid supplementation (EPA and DHA) on the transcription of mRNA in T CD4+ lymphocytes subsets. Data shown in mean ± SEM.

We analyzed IL-6 and TNF-α as markers for overall inflammation and, in each of the T CD4+ lymphocyte subsets, one “master” transcription factor and one signature cytokine: TBX21 and IFNG in Th1; GATA3 and IL-4 in Th2; and FOXP3, and IL-10 in Treg cells. At time 0, the level of transcription (mRNA) of all genes analyzed were higher in the group with obesity compared with the control group, ranging between 3.5-times of TNF-α and 18.7-times of IL-4 (Figure 5). Importantly, the mRNA levels of all these genes did not change in the control group during or after supplementation (data not shown). Therefore, for comparison purposes, the level of transcription of each gene was normalised against time 0 in the control group. In the group with obesity, the mRNA levels of all genes studied decreased to levels comparable to the healthy group after the first month of supplementation and remained low within the entire duration of the study, even 30 days after cessation (Figure 6).

## Discussion

**Figure 7:**
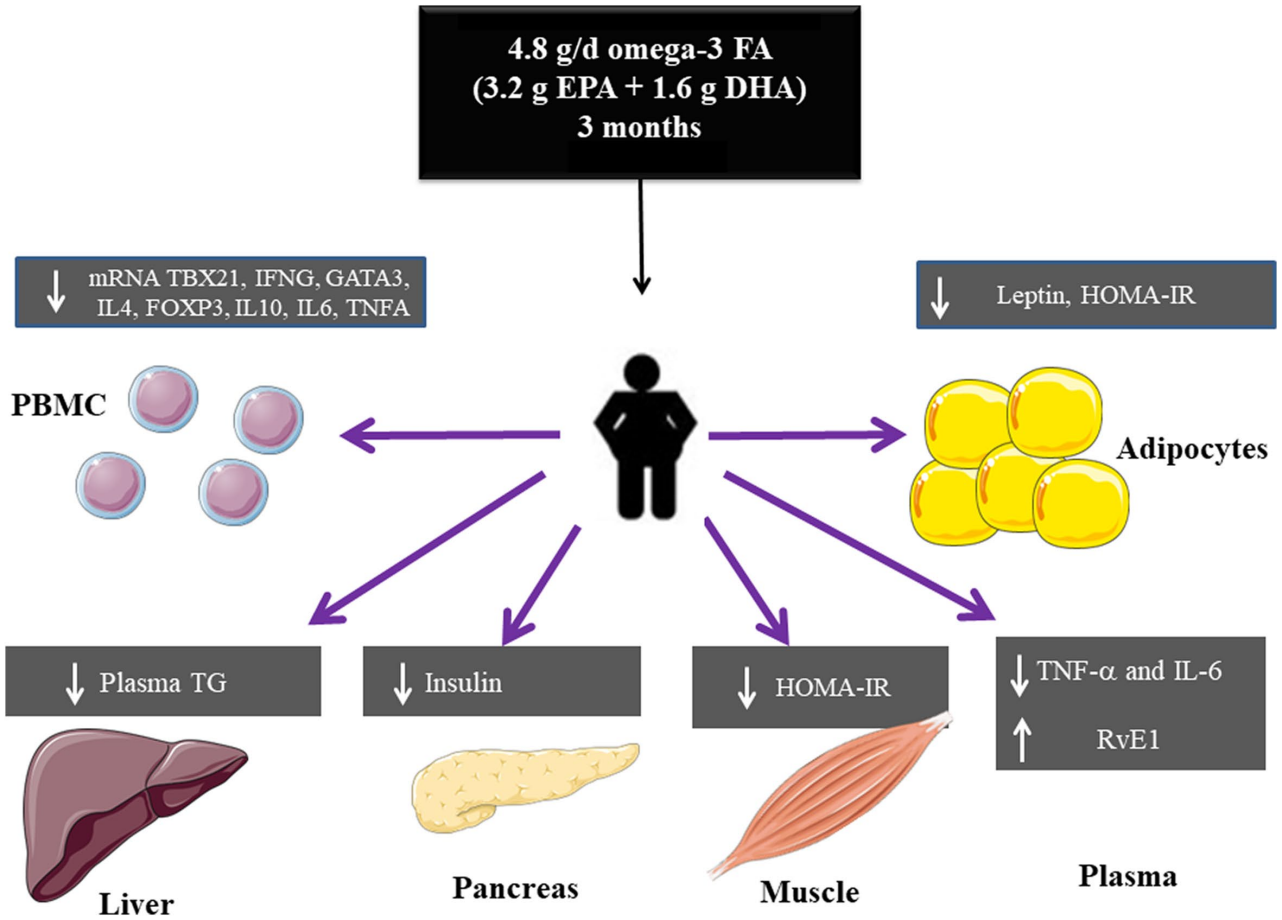
Summary of the effects of high dose omega-3 FA on metabolic and inflammatory markers in obesity.

In this study we assessed the effect of high-dose omega-3 FA, supplementation, 4.8 g/day (3.2 g of EPA plus 1.6 g of DHA), for 3 months in the form of fish oil capsules, on metabolic and inflammatory markers in Mexican patients with obesity and healthy volunteers. The main results are summarized in Figure 7.

This study has several strengths. First, all participants were carefully selected, particularly the patients with obesity. Therefore the results reported here pertain to patients without chronic diseases associated with obesity. Second, the inclusion of clinically healthy volunteers (control group) instead of a placebo group was a point of strength, because they were reference to contrast the differences in metabolic and inflammatory markers against the group with obesity: extreme phenotypes (healthy versus obese). Lastly, we included a 1 month period without treatment to determine the long-term effect of supplementation on the metabolic and inflammatory markers in both groups.

The fish oil supplement used as source of EPA and DHA, provided extra 72 kcal per day (8 grams, 1 gram/capsule), it was well accepted with a high compliance (over 92 percent). As previously stated, regardless of their nutritional status, none of the participants presented with changes in body weight or composition (fat mass and lean mass) during the duration of the study (Supplementary Table S2).

Of note, there were differences between the group with obesity and control group at baseline. The group with obesity showed higher values of metabolic and inflammatory markers. However most of these values were within normal range or slightly higher, except for TG, HDL-C, HOMA-IR, leptin, and A/L ratio which were not within the normal range. However, it is important to mention that these values did not indicate presence of type 2 diabetes mellitus, hypertension, or other diseases.

In Mexico, the total daily intake of omega-3 FA has been estimated to be 0.3 grams per day in adults (24), which is very low compared with the suggested 2.0 grams per day of EPA plus DHA set by the Food and Agriculture Organization for adults worldwide (25). It has been shown that plasma levels of EPA and DHA reflected a short-term and dose-dependent intake (13), whereas erythrocytes fatty acids composition indicated chronic consumption. However, erythrocytes might be better indicators for longer periods of supplementation (longer than 3 months due to their turnover period). In this study, we evaluated plasma concentration of fatty acids to determine acute changes (26).

The low intake of omega-3 FA by the Mexican population, regardless of their nutritional or inflammatory status, was confirmed by a high omega-3/omega-6 ratio with values of 12.4 and 10.3 and AA/EPA ratio with values of 11.1 and 13.6 in the control and obesity groups, respectively, at time 0 (Supplementary Table S3 and Figure 3). To our best knowledge, this is the first time that the AA/EPA ratio in plasma to be reported in a Mexican cohort. The AA/EPA values reported in this study (above 10), are similar to those reported in United States, Canada, Greece, Italy, Spain, and the Netherlands regarding individuals who consumed “westernised diets.” These diets might activate the immune system, because they are high in energy-dense nutrients and refined sugars, red meat, high-fat dairy products, together with low amounts of vegetables, fruits, whole grains, fish, or nuts (27). In other regions of the world where the consumption of fish and fish products is high, the AA/EPA ratio is low (below 5), like Canadian Inuits, US-Alaskans, Japan, Papua-New Guinea, Korea, Russia, Iceland, and Norway, just to mention a few (28).

In our study, the AA/EPA ratio was reduced in both groups after the first month of supplementation to values of 1.2 and 3.2 in the control and obesity groups, respectively (Figure 3) and remained low for the duration of the treatment. A previous study has shown that the reduction in AA/EPA ratio was dose-dependent (13). After supplementation cessation (time 4), the AA/EPA ratio in the group with obesity returned to its initial value, whereas in the control group remained low, without significant difference from its starting value. Although the omega-6/omega-3 decreased to levels lower than 4 during the supplementation period, which is the recommended value, it returned to basal levels at time 4 (29).

In this study, we observed that, in the control group, no RvE1 was detectable in plasma. In contrast, the group with obesity included three participants with detectable levels at time 0. In addition, after one-month supplementation, the RvE1 levels non-significantly increased in both groups. Importantly, our data of RvE1 need to be taken with caution due to the quantification method used in this study, which might not be a reliable method for quantifying this pro-resolving mediator. The required sensitivity and selectivity for these types of mediators apparently could only be achieved using chromatography coupled with mass spectrometry (30, 31).

### Effect of the omega-3 FA supplementation on the metabolism of glucose and lipids

Regardless of the nutritional status, a dose of 4.8 g/d of EPA plus DHA in the form of fish oil supplement revealed beneficial effects on the metabolism of glucose in all participants. These effects pertain only to insulin, due to a reduction in its concentration (fasting levels) and resistance (lower HOMA-IR values), but not in serum glucose.

The clinical impact of these effects was more significant in the group of patients with obesity. Because, despite their glucose levels were higher (but still within the normal range) and did not change with supplementation, the fasting levels of insulin and the HOMA-IR values indicated reductions in insulin resistance and, more importantly HOMA-IR reached values below the cut-off point of 2.5 (16). Moreover, both insulin and HOMA-IR decreased in the entire duration of the study and remained low even 1 month after supplementation cessation. Despite promising, these results warrant further studies, for instance oral-glucose tolerance test to determine the glycemic and insulinemic responses or hyperinsulinemic-euglycemic clamp, the “gold standard” to assess insulin action. Only these tests would provide unequivocal data on the real effect of omega-3 FA supplementation on glucose metabolism in patients with obesity.

Our results also showed improvements in some parameters of lipid metabolism: LDL-C did not change with supplementation, whereas HDL-C and TG did. Although differences in the levels HDL-C and TG did not reach statistical significance, there was a tendency of an increase in HDL-C concentration in both groups. This increase might have clinical relevance, because even a small increase of 1 mg/dL in HDL-C has been found to be associated with a 6 percent decrease in cardiovascular risk (32).

Reduction in serum and liver TG is the best-known action of EPA and DHA, by a mechanism still not completely explained (33, 34). In our study, we also observed that the supplement of fish oil caused a (not significantly) reduction in serum TG in both groups during supplementation, which returned to its initial levels after cessation. This is particularly relevant in the group with obesity, since TG levels decreased from an average of 191 mg/dL to 148 mg/dL, which was within the normal range and associated with reduced cardiovascular events. The TG/HDL-C has been used as a predictable marker for insulin resistance and has been associated with higher risk of cardiovascular diseases (35). In the study, we observed a very high TG/HDL-C ratio in the group with obesity, which non-statistically decreased during the intervention phase and returned to its initial value after cessation.

### Effect of the omega-3 FA supplementation on the inflammatory status

Omega-3 FA supplementation decreased TNF-α levels in both groups, other inflammatory markers improved after supplementation without statistical difference (IL-6, IL-10, and TGF-β). However, leukocytes, ESR, IL-8, adiponectin (total and its isoforms), and A/L ratio remained unaltered.

TNF-α is an inflammatory protein synthesised by immune and adipose cells and promotes insulin resistance by alterations in the insulin receptor signaling pathway (36). In addition, TNF-α induces inflammation *via* the NF-kB pathway producing an increase in several cytokines including IL-6 and others. IL-6 is produced by many cell types (monocytes, fibroblasts, endothelial) and together with TNF-α acts on different tissues causing inflammation.

In the present study, omega-3 FA supplementation decreased plasma levels of TNF-α in both groups (Table 2) and mRNA of TNF-α and IL-6 (Figure 6) in PBMC. These effects could be explained by the interactions of omega-3 FA with the surface receptor FFAR4 (GPR120) (37), or their interactions with PPARγ, which result in NF-kB signaling inhibition (37, 38).

Of note, although plasma TNF-α was identical in both groups at baseline (Table 1) as previously described (39), there was no insulin resistance in the control group. In a previous study, mice models with TNF-α genetic deletion did not show improvement in insulin resistance (40). Therefore, we suggest that, besides TNF-α, there should be other factors in the group with obesity involved in insulin resistance, like high levels of leptin, along with low levels of adiponectin and IL-10, which may interfere with inflammation control and insulin sensitivity. The significant reduction in plasma TNF-α in both groups, was not paralleled with its mRNA transcript at time 4, the latter remained downregulated even 1 month after cessation. A previous study including healthy volunteers reported that consumption of 18 grams of fish oil (4.6 g of EPA plus DHA) reduced the synthesis *ex vivo* of TNF-α and IL-1β in PBMC even 10 weeks after cessation (41). This result is similar to our findings regarding mRNA transcripts in PBMC and may be partially explained by the incorporation of EPA and DHA into cell membranes (42) and to the longer turnover of lymphoid cells (43). The return of plasma TNF-α to its initial levels after supplementation cessation could also be the result of its production by other cells.

One of the most common features of inflammation is leukocytosis (2). We also observed mild leukocytosis in the group with obesity which was still within the normal range. This increase in leukocytes could be partly due to increases in CD4^+^ T lymphocytes, including Treg cells that we (44) and others (45) have previously reported. The higher transcription of mRNA FOXP3 and IL-10 (Treg markers), accompanied by a high transcription of TBX21 and IFNG (Th1 markers), GATA3 and IL-4 (Th2 markers) (Figure 5) indicated the inflammatory status in the group with obesity and an altered Treg function. However, Treg functionality has not been assessed in individuals with obesity. Of note, our results in PBMC are similar to those reported in subcutaneous (46, 47) and visceral (47) adipose tissue in patients with obesity. These findings also revealed that PBMC could be used as a biomarker of adipose tissue inflammation (48).

The fish oil supplement did not lead to changes in total leukocytes. However, it had profound effects on mRNA transcript levels of CD4+ T lymphocyte subsets, TBX21 and IFNG in Th1 cells, GATA3 and IL-4 in Th2 cells and FOXP3, and IL-10 in Treg cells. This downregulation was observed during the entire supplementation phase and remained even 1 month after cessation (Figure 6). These results indicated that a high dose of omega-3 FA may could modulate T CD4+ lymphocyte subsets activation, differentiation, and proliferation (7, 9, 49).

On the other hand, increments in the levels of IL-10 and TGF-β, cytokines synthesised by Treg and monocytes M2 might be a part of the modulatory and anti-inflammatory effect of omega-3 FA.

Controversial effects have been reported after omega-3 FA supplementation. This might be explained by the heterogeneity in the methodology used in different studies including supplementation dose, study group, and time of supplementation (6, 11). Most of the studies of omega-3 FA supplementation, which showed anti-inflammatory effects, used doses over 3 g/d (6). In our study, an anti-inflammatory effect of high-dose omega-3 FA was observed. However, a high-dose of EPA (4 g/d) in individuals above 45 years of age with hypercholesterolemia has shown no important effects on inflammatory markers (11). In our study we found an anti-inflammatory effect using a high-dose omega-3 FA (4.8 g/d). However, the population in this present study included women between 18–45 years of age, with obesity and without other chronic diseases.

One of the limitations of this study was the sample size. However the statistical power of the sample was 82%. Although the lack of a placebo group is another limitation, each participant was evaluated versus a control. Therefore, whether the results of this study can be applied to other groups remains unexplored. Another limitation of the study was the lack of biopsies to analyze CD4^+^ T cells in adipose tissue.

Improvements in inflammation and insulin resistance by a high-dose omega-3 FA *in vivo* in the participants with obesity, observed in this study, could be clinically important because overall mortality is more related to inflammation and hyperinsulinemia rather than to obesity (50). Therefore, omega-3 FA supplementation should be an adjuvant therapy in inflammatory conditions like obesity.

## Conclusion

Supplementation with a high dose of omega-3 FA could modulate metabolic alterations and inflammation in patients with obesity. There were differential effects of the omega-3 FA supplementation between inflammatory and metabolic markers. The beneficial effects were ephemeral regarding most metabolic markers. In contrast, most changes in inflammatory markers remained reduced after cessation.

## Data availability statement

The original contributions presented in the study are included in the article/Supplementary files, further inquiries can be directed to the corresponding author.

## Ethics statement

The studies involving human participants were reviewed and approved. This study was approved by the Ethics Committee of the INCMNSZ. This study is registered at Clinical trials with the ID NCT05219890. The patients/participants provided their written informed consent to participate in this study.

## Author contributions

AB-M participated in the recruitment, follow-up of the participants during treatment, evaluated mRNA expression of interest genes, inflammatory markers, and wrote the manuscript. MG-C and AF-L quantified metabolic markers. SC-D quantified fatty acids in plasma. JG, CA, and HB participated in the design of the study. MP was the Inflammation Research Foundation contact and provided the EPA and DHA supplement. BS was the Inflammation Research Foundation contact and provided the EPA and DHA supplements and participated in the design of the study. FEG conceived the study, participated in its design and coordination, obtained funds, and wrote and reviewed the final version of the manuscript. All authors contributed to the article and approved the submitted version.

## Funding

This study was partially funded by CONACYT (grant CF-191983 to FEG). Angelica Borja-Magno is a doctoral student in Programa de Doctorado en Ciencias Biomédicas, Universidad Nacional Autónoma de México (UNAM) and has received CONACyT fellowship 607487.

## Conflict of interest

MP and BS are employees of Zone Labs, Inc., a medical food company that produces omega-3 fatty acid products.

The remaining authors declare that the research was conducted in the absence of any commercial or financial relationships that could be construed as a potential conflict of interest.

## Publisher’s note

All claims expressed in this article are solely those of the authors and do not necessarily represent those of their affiliated organizations, or those of the publisher, the editors and the reviewers. Any product that may be evaluated in this article, or claim that may be made by its manufacturer, is not guaranteed or endorsed by the publisher.
