# Supplementary material for: Differential effects of high dose omega-3 fatty acids on metabolism and inflammation in patients with obesity: eicosapentaenoic and docosahexaenoic acid supplementation
Source: Front Nutr. 2023 May 5;10:1156995. doi: 10.3389/fnut.2023.1156995 (PMC10196397; doi:10.3389/fnut.2023.1156995)
Supplement: Supplementary file 1 [file Data_Sheet_1.docx]

***Supplementary Material***

**Differential effects of high dose of omega-3 fatty acids on metabolism and inflammation in patients with obesity: eicosapentaenoic and docosahexaenoic acid supplementation**

**Angélica Borja-Magno, MSc^1^, Martha Guevara-Cruz, MD, PhD^1^, Adriana Flores-López, MSc^2^, Silvia Carrillo-Domínguez, PhD^3^, Julio Granados MD^4^, Clorinda Arias, MD, PhD^5^, Mary Perry, MS, RD, LDN^6^, Barry Sears, PhD^6^, Hector Bourges MD, PhD^7^, and F. Enrique Gómez, PhD^1^*.**

***Correspondence:**

F. Enrique Gómez

Departamento de Fisiología de la Nutrición

Instituto Nacional de Ciencias Médicas y Nutrición Salvador Zubirán

Av. Vasco de Quiroga No.15. Colonia: Belisario Domínguez Sección XVI, Delegación Tlalpan. C.P:14080, Ciudad de México, México.

Email: francisco.gomezr@incmnsz.mx

Supplementary Table 1. Sequences of the primers used for the analysis of mRNA levels in T CD4+ lymphocyte subsets

Supplementary Table 2. Effect of omega-3 fatty acid supplementation (EPA and DHA) on weight, body composition and on blood pressure.

Supplementary Table 3. Time-course effect of omega-3 fatty acid supplementation (EPA and DHA) on plasma fatty acid levels.

| **Supplementary Table 1. Sequences of the primers used for the analysis of mRNA levels in T CD4+ lymphocyte subsets** | | | |
| --- | --- | --- | --- |
| Gene | Forward primer | Reverse primer | Amplicon size (bp) |
| TNFA | 5´ CTGGGCAGGTCTACTTTGGG 3´ | 5´CTGGAGGCCCCAGTTTGAAT 3´ | 272 |
| IL6 | 5´ CCACCGGGAACGAAAGAGAA 3´ | 5´GAGAAGGCAACTGGACCGAA 3´ | 92 |
| TBX21 | 5´ GTCGGTGTCCTCCAACCTAA 3´ | 5´CCCTTGGTGTGGACTGAGAT 3´ | 245 |
| IFNG | 5´ ATATTGCAGGCAGGACAACC 3´ | 5´TCATCCAAGTGATGGCTGAA 3´ | 116 |
| GATA3 | 5´ TTTTTCGGTTTCTGGTCTGG 3´ | 5´CTCATTAAGCCCAAGCGAAG 3´ | 205 |
| IL4 | 5´ CCAACGTACTCTGGTTGGCT 3´ | 5´GCACCGAGTTGACCGTAACA 3´ | 258 |
| FOXP3 | 5´ ATTGAGTGTCCGCTGCTTCT 3´ | 5´TCCCAGAGTTCCTCCACAAC 3´ | 122 |
| IL10 | 5´ TCTCGGAGATCTCGAAGCAT 3´ | 5´TGCAAAACCAAACCACAAGA 3´ | 176 |
| 18s rRNA | 5´ CCTCCAATGGATCCTCGTTA 3´ | 5´AAACGGCTACCACATCCAAG 3´ | 155 |

| **Supplementary Table 2. Effect of omega-3 fatty acid supplementation (EPA and DHA) on weight, body composition and on blood pressure.** | | | | | | |
| --- | --- | --- | --- | --- | --- | --- |
|  | | Time 0 | Time 3 | *p* value^†^ | *P* value₸ | *P* value¥ |
| ANTHROPOMETRY AND BODY COMPOSITION | | | | |  |  |
| Weight (kg) | Control | 56.3 ± 9.0 | 57.2 ± 5.7 | 0.11 | 0.0001 | 0.92 |
|  | Obesity | 102.1 ± 15.4 | 102.6 ± 14.4 |  |  |  |
| BMI (kg/m^2^) | Control | 21.7 ± 2.2 | 21.6 ± 2.2 | 0.10 | 0.0001 | 0.81 |
|  | Obesity | 39.8 ± 5.5 | 39.7 ± 5.4 |  |  |  |
| Fat mass (kg) | Control | 15.0 ± 3.9 | 15.8 ± 3.3 | 0.19 | 0.0001 | 0.61 |
|  | Obesity | 42.7 ± 7.8 | 41.7 ±7.4 |  |  |  |
| Fat mass (%) | Control | 25.5 ± 5.1 | 26.7 ± 4.4 | 0.96 | 0.0001 | 0.39 |
|  | Obesity | 41.9 ± 3.6 | 40.9 ± 3.9 |  |  |  |
| Lean body mass (kg) | Control | 41.8 ± 3.6 | 43.6 ± 7.9 | 0.89 | 0.0001 | 0.90 |
|  | Obesity | 59.3 ± 9.8 | 60.3 ± 10.8 |  |  |  |
| Lean body mass (%) | Control | 74.4 ± 5.3 | 73.3 ± 7.2 | 0.90 | 0.0001 | 0.83 |
|  | Obesity | 58.1 ± 3.6 | 59.1 ± 4.9 |  |  |  |
| BLOOD PRESSURE | | | | | | |
| Systolic BP (mm Hg) | Control | 106.0 ± 8.0 | 108.0 ± 9.0 | 0.25 | 0.0001 | 0.76 |
|  | Obesity | 123.0 ± 8.0 | 123.0 ± 6.0 |  |  |  |
| Diastolic BP (mm Hg) | Control | 67.0 ± 6.0 | 65.0 ± 7.0 | 0.20 | 0.0001 | 0.37 |
|  | Obesity | 78.0 ± 9.0 | 81.0 ± 12.0 |  |  |  |
| Mean ± SD. Two-way ANOVA for repeated measures.  † Time factor (comparisons within groups *vs* time 0).  ₸ Group factor (comparison between groups obesity *vs* control)  ¥ Interaction (effect of group and time on the metabolic or inflammatory marker)  * *p*<0.05. | | | | | | |

| Supplementary Table 3. Time-course effect of omega-3 fatty acid supplementation (EPA and DHA) on plasma fatty acid levels. | | | | | | | | | |
| --- | --- | --- | --- | --- | --- | --- | --- | --- | --- |
|  | | Time 0 | Time 1 | Time 2 | Time 3 | Time 4 | *p* value^†^ | *P* value₸ | *P* value¥ |
| Lauric acid (12:0) (mM) | Control | 0.1 ± 0.1 | 0.08 ± 0.06 | 0.05 ± 0.04 | 0.04 ± 0.05 | 0.1 ± 0.07 | 0.10 | 0.06 | 0.11 |
|  | Obesity | 0.05 ± 0.07 | 0.03 ± 0.02 | 0.1 ± 0.1 | 0.02 ± 0.03 | 0.05 ± 0.08 |  |  |  |
| Myristic acid (14:0) (mM) | Control | 1.4 ± 1.6 | 0.5 ± 0.2 | 0.4 ± 0.1 | 0.4 ± 0.2 | 0.7 ± 0.2 | 0.62 | 0.17 | 0.46 |
|  | Obesity | 1.0 ± 1.1 | 1.0 ± 1.5 | 1.2 ± 2.0 | 1.0 ± 1.4 | 0.7 ± 0.6 |  |  |  |
| Palmitic acid (16:0) (mM) | Control | 33.3 ± 42.7 | 10.5 ± 2.7 | 8.9 ± 1.9 | 9.1 ± 3.8 | 12.5 ± 4.3 | 0.47 | 0.13 | 0.59 |
|  | Obesity | 27.5 ± 37.8 | 27.3 ± 40.3 | 28.9 ± 48.6 | 26.0 ± 45.0 | 13.0 ± 9.8 |  |  |  |
| Stearic acid (18:0) (mM) | Control | 23.2 ± 30.4 | 8.0 ± 2.1 | 8.4 ± 4.8 | 6.5 ± 1.9 | 8.8 ± 2.3 | 0.31 | 0.26 | 0.51 |
|  | Obesity | 18.6 ± 27.6 | 17.1 ± 24.1 | 17.5 ± 27.5 | 16.2 ± 25.1 | 8.6 ± 11.0 |  |  |  |
| Palmitoleic acid (16:1 n-7) (mM) | Control | 2.3 ± 3.2 | 0.4 ± 0.2 | 0.4 ± 0.1 | 0.6 ± 0.7 | 0.7 ± 0.5 | 0.47 | 0.13 | 0.59 |
|  | Obesity | 2.5 ± 3.5 | 2.2 ± 3.6 | 2.4 ± 5.0 | 1.8 ± 2.9 | 1.2 ± 0.4 |  |  |  |
| Oleic acid (18:1 n-9) (mM) | Control | 22.9 ± 28.8 | 6.9 ± 2.2 | 5.8 ± 1.5 | 6.5 ± 4.0 | 8.8 ± 3.0 | 0.51 | 0.08 | 0.73 |
|  | Obesity | 22.7 ± 32.7 | 19.1 ± 27.6 | 21.2 ± 35.6 | 22.4 ± 43.7 | 9.6 ± 4.8 |  |  |  |
| Linoleic acid (18:2 n-6) (mM) | Control | 36.7 ± 50.5 | 9.1 ± 2.2 | 8.0 ± 2.1 | 8.8 ± 3.2 | 11.7 ± 3.0 | 0.43 | 0.14 | 0.50 |
|  | Obesity | 28.7 ± 40.6 | 30.3 ± 47.0 | 32.6 ± 57.2 | 26.6 ± 52.8 | 10.2 ± 4.7 |  |  |  |
| α-Linolenic acid (18:2 n-3) (mM) | Control | 0.7 ± 1.0 | 0.2 ± 0.1 | 0.2 ± 0.1 | 0.2 ± 0.1 | 0.3 ± 0.1 | 0.57 | 0.03 | 0.57 |
|  | Obesity | 0.7 ± 0.8 | 0.8 ± 1.2 | 0.8 ± 1.3 | 0.7 ± 1.3 | 0.2 ± 0.2 |  |  |  |
| Arachidonic acid (20:4 n-6) (mM) | Control | 6.2 ± 8.8 | 1.7 ± 0.8 | 1.7 ± 0.5 | 1.6 ± 0.7 | 1.9 ± 0.6 | 0.33 | 0.18 | 0.48 |
|  | Obesity | 4.7 ± 6.6 | 5.6 ± 8.8 | 5.3 ± 8.2 | 3.7 ± 6.1 | 1.6 ± 0.6 |  |  |  |
| Eicosapentaenoic acid (20:5 n-3) (mM) | Control | 0.5 ± 0.8 | 1.7 ± 0.6 | 3.4 ± 6.8 | 0.7 ± 0.6 | 1.2 ± 2.2 | 0.05 | 0.70 | 0.51 |
|  | Obesity | 0.4 ± 0.5 | 4.3 ± 7.8 | 2.7 ± 3.2 | 1.5 ± 0.8 | 0.2 ± 0.3 |  |  |  |
| Docosahexaenoic acid (22:6 n-3) (mM) | Control | 1.4 ± 2.0 | 1.0 ± 0.3 | 0.9 ± 0.2 | 0.7 ± 0.3 | 0.9 ± 0.8 |  |  |  |
|  | Obesity | 0.9 ± 1.3 | 2.7 ± 4.1 | 2.3 ± 3.5 | 1.8 ± 2.7 | 0.5 ± 0.2 | 0.55 | 0.12 | 0.30 |
| PUFA / SFA ratio | Control | 0.7 ± 0.5 | 0.7 ± 0.7 | 0.7 ± 0.5 | 0.7 ± 0.5 | 0.8 ± 0.5 | 0.91 | 0.06 | 0.99 |
|  | Obesity | 0.5 ± 0.3 | 0.6 ± 0.3 | 0.5 ± 0.4 | 0.5 ± 0.3 | 0.7 ± 0.2 |  |  |  |
| Omega-6/ Omega-3 ratio | Control | 12.4 ± 4.2 | 3.7 ± 1.8* | 4.4 ± 3.5* | 6.5 ± 4.6* | 7.6 ± 6.0* | **0.001** | 0.95 | 0.26 |
|  | Obesity | 10.3 ± 2.6 | 5.1 ± 2.3* | 5.2 ± 2.6* | 4.9 ± 2.4* | 9.0 ± 1.8 |  |  |  |
| EPA + DHA | Control | 1.9 ± 2.8 | 2.6 ± 1.0 | 4.1 ± 6.8 | 1.52 ± 0.7 | 2.5 ± 2.5 | 0.08 | 0.60 | 0.34 |
|  | Obesity | 1.2 ± 1.8 | 6.8 ± 11.9 | 4.9 ± 6.7 | 2.2 ± 1.4 | 0.6 ± 0.4 |  |  |  |
| Mean ± SD. Two-way ANOVA for repeated measures.  † Time factor (comparisons within groups *vs* time 0).  ₸ Group factor (comparison between groups obesity *vs* control)  ¥ Interaction (effect of group and time on the metabolic or inflammatory marker  * Statistical significance *p*<0.05.  PUFA: Polyunsaturated fatty acids. SFA: Saturated fatty acids | | | | | | | | | |
